# Development and validation of a novel multivariate risk score to guide biopsy decision for the diagnosis of clinically significant prostate cancer

**DOI:** 10.1002/bco2.8

**Published:** 2020-03-12

**Authors:** Helmut Klocker, Bruno Golding, Stephan Weber, Eberhard Steiner, Pierre Tennstedt, Thomas Keller, Ralph Schiess, Silke Gillessen, Wolfgang Horninger, Thomas Steuber

**Affiliations:** ^1^ Department of Urology Medical University Innsbruck Innsbruck Austria; ^2^ ProteoMediX AG Schlieren Switzerland; ^3^ ACOMED Statistics Leipzig Germany; ^4^ Martini Clinic Prostate Cancer Center University Hospital Hamburg‐Eppendorf Hamburg Germany; ^5^ Medical Oncology Department Oncology Institute of Southern Switzerland Bellinzona Switzerland

**Keywords:** biopsy, cancer significance, cathepsin D, CTSD, free PSA, grade group, Proclarix, prostate cancer, THBS1, thrombospondin‐1, total PSA

## Abstract

**Objectives:**

Selecting patients suspected of having prostate cancer (PCa) for a prostate biopsy remains a challenge. Prostate‐specific antigen (PSA)‐based testing is hampered by its low specificity that often leads to negative biopsy results or detection of clinically insignificant cancers, especially in the 2‐10 ng/mL range. The objective was to evaluate a novel diagnostic test called Proclarix incorporating thrombospondin‐1 and cathepsin D alongside total and free PSA as well as age for predicting clinically significant PCa.

**Patients and methods:**

The test was developed following a retrospective study design using biobanked samples of 955 men from two reference centres. A multivariate approach was used for model development followed by validation to discriminate significant (grade group ≥2) from insignificant or no cancer at biopsy. The test specificity, positive predictive value (PPV) and negative predictive value (NPV) at a fixed sensitivity of 90% were compared to percent free PSA (%fPSA) alone. The number of avoidable prostate biopsies deemed to be representative of clinical utility was also assessed.

**Results:**

In the targeted patient population, the test displayed increased diagnostic accuracy compared to %fPSA alone. Application of the established model on 955 patients at a fixed sensitivity of 90% for significant disease resulted in a specificity of 43%, NPV of 95% and a PPV of 25%. This is in comparison to a specificity of 17%, NPV of 89% and PPV of 19% for %fPSA alone and had the potential to reduce the total number of biopsies needed to identify clinically significant cancer. Further, the test score correlated with significance of cancer assessed on prostate biopsy.

**Conclusions:**

The Proclarix test can be used as an aid in the decision‐making process if to biopsy men in this challenging patient population. The use of the test could reduce the number of biopsies performed avoiding invasive procedures, anxiety, discomfort, pain and complications.

## 1 INTRODUCTION

Selecting patients suspected of having clinically significant prostate cancer (PCa) **(**ISUP grade group GG ≥ 2) for a prostate biopsy remains a challenge despite the growing number of available diagnostic tools. The challenge to orient biopsy decision making is especially present when evaluating patients within the “prostate‐specific antigen (PSA) grey zone” of total PSA (tPSA) 4‐10 ng/mL^1^ or 2‐10 ng/mL,^2^, ^3^ a growing cohort of patients with an ageing population, where test performance can vary. The decision to biopsy is further compounded when considering the influence of prostate volume,^4^ family history,^5^ prior biopsy status^6^ and digital rectal exam (DRE) status.^7^

The diagnostic tools currently available are generally PSA based, using other forms of PSA, different molecular markers or mathematical combinations of such markers.^8^ While they represent an improvement, their performance levels vary depending on the validation cohort used and the intended target population in terms of PSA range, prostate volume and DRE characteristics. PSA is historically and currently the most frequently used marker but it is not cancer‐specific^9^ and its low specificity leads to over diagnosis.^10^ In consequence, depending on the specific cohort,only about 25% up to a maximum of 60% of men with tPSA values in the 4‐10 ng/mL range have a positive biopsy.^11^ Lower cut‐offs, for example, 2 ng/mL or age‐specific cut‐offs can improve sensitivity, but sacrifice specificity.^12^ The ratio of free PSA (fPSA) to tPSA (percent free PSA [%fPSA]) has also been shown to improve test performance^11^ but has limitations as %fPSA both increases with age as well as with prostate size and yields improved results only in patients with small prostates (<40 mL).^13^, ^14^

We have previously shown in one of the most challenging subsets of subjects presenting with a tPSA of 2‐10 ng/mL, prostate volume ≥35 mL, no prior history of PCa and a normal DRE, that the combined measurement of two novel glycoproteins thrombospondin‐1 (THBS1) and cathepsin D (CTSD) can improve the identification of clinically significant PCa.^15^ Based on these results we have developed a new test named Proclarix. This test incorporates THBS1 and CTSD with patient age, tPSA and %fPSA values into a dedicated algorithm and provides a risk score that corresponds to the probability of detecting clinically significant PCa on biopsy.

The purpose of this study was to validate the performance of Proclarix including the 5‐parameter multivariate logistic regression algorithm, as compared to %fPSA alone in discriminating no cancer and GG < 2 versus GG ≥ 2.

## 2 MATERIAL AND METHODS

### 2.1 Study design

This was a prospectively planned, retrospective, blinded, 2‐center study using biobanked samples to establish and validate the use of the test in identifying clinically significant PCa. Samples were blinded to experimenters and were only unblinded once measurements were complete. A formal sample size calculation was not performed, however, to select predictors to forecast a binary outcome from *k* variables, it is proposed that there would need to be *k* × 10 to *k* × 20 cases in the smaller group,^16^ whereas Steyerberg et al^17^ recommends *k* × 50 cases and mentions a lower limit is *k* × 10 cases. Considering five biomarkers, a sample size of 100 results for the smaller group (positive cases) is deemed sufficient to meet the proposed requirements.

### 2.2 Study population

The study population consisted of biobanked samples from two centres (474 individual samples from the Martini‐Klinik, University Hospital Hamburg‐Eppendorf, Hamburg, Germany and 481 samples from Medical University Innsbruck, Innsbruck, Austria. The following inclusion and exclusion criteria were applied to both cohorts. Patient samples collected before biopsy from patients with a tPSA 2‐10 ng/mL, >18 years old, normal DRE, prostate volume ≥35 mL determined by transrectal ultrasound (TRUS) were included in this study. Samples from patients with a history of prior pharmacological treatment for benign prostatic hyperplasia (BPH), PCa or prostatitis were not included. In addition, haemolytic, icteric or lipemic samples and samples with freeze thaw cycles ≥3 and missing required clinical data were excluded from the study. For all patients results for TRUS‐guided 10‐12 core prostate biopsy were available. The study population is comprised of samples collected consecutively and independently of biopsy results, thus representing the prevalence and spectrum of the population of the centres. The use of biobanked material was approved by the local ethics committees and all patients had given a general written informed consent for storage and future studies of their samples.

### 2.3 Assay methods

The Proclarix test is comprised of two quantitative Enzyme‐linked Immunosorbent Assays (ELISA) that measure the concentration of THBS1 and CTSD in human serum. A dedicated software integrates the values for THBS1 and CTSD, age, tPSA and fPSA (from third party manufacturers) to calculate a risk score. The ELISAs along with the software form the CE marked Proclarix test. The ELISA kits used in this study were derived from two different manufacturing lots to which samples were randomly assigned. If during measuring samples were outside of the measuring range of the assay, samples were re‐diluted and remeasured so that all subjects in the study population had a test result. Serum tPSA and fPSA were re‐analysed for Hamburg samples using the ADVIA Centaur immunoassay system (Siemens Healthcare) to calculate %fPSA. Available PSA values of samples from Innsbruck were obtained from an Elecsys system (Roche Diagnostics). Due to known variations for tPSA and fPSA measurements between analysis kits from different manufacturers,^18^ tPSA and fPSA values were normalised to data generated on the Elecsys system. This normalisation was based on median values derived across multiple sites from INSTAND e.V. ring study data; the normalised data were then used for the model development (https://rv-online.instandev.de/index.shtml accessed: 17 April 2019).

### 2.4 Statistical methods for test development and validation

#### 2.4.1 Model development

An initial feature selection was performed on the dataset to obtain the best possible model to predict the outcome of clinically significant PCa (GG ≥ 2). This resulted in a final mathematical biomarker model incorporating THBS1, CTSD, tPSA, %fPSA and age that was subsequently established using all 955 samples. The “risk score” is derived from the regression analysis and is represented as a percentage scale from 0%‐100% indicating the risk of significant PCa. The cut‐off was set at a sensitivity of 90%, no further model recalibration was performed.

#### 2.4.2 Model assessment

The mathematical biomarker model was validated using a split sample approach, where the complete dataset has been divided into a training and validation dataset in a 100:70 ratio with balanced prevalence. Logistic regression parameter estimates and a cut‐off for the linear predictor, referring to 90% sensitivity selected for a 10% false negative rate, as obtained from training cohort's analyses, have been applied to samples in the validation set in order to assess the model's suitability. In total, the model validation was based upon 1000 independent sets of training and validation sets. In order to evaluate the performance of the developed model,^19^ the results derived from the validation datasets have been reported. This has been evaluated for the entire dataset as well as for each of the 1000 random split samples by calculating specificity, negative predictive value (NPV) and positive predictive value (PPV) at 90% (training set) sensitivity, using the model on one side and %fPSA on the other as predictors. The difference in specificities between the test and %fPSA in the complete dataset were assessed using McNemar Test. All analyses were performed with SAS (Version 9.4) or R (3.2.3). The impact of the index‐test, in this case the biomarker model, was compared to the reference test of %fPSA alone and was selected as the current recommendation to avoid unnecessary biopsies is the use of an additional serum test such as %fPSA.^3^

## 3 RESULTS

### 3.1 Study population

Patient characteristics are summarised in Table 1. Overall, the total cohort comprised of 170 clinically significant PCa (GG ≥ 2) (17.8%) and 785 negative or GG < 2 PCa samples. This represents 106 clinically significant PCa (22.4% of total cohort) in Hamburg and 64 clinically significant PCa (13.3% of total cohort) in Innsbruck.

**Table 1:** Overview of patient characteristics by cohort

Characteristic	Total	Innsbruck cohort	Hamburg cohort
Patients, n	955	481	474
Age range, year (mean)	42‐85 (63)	44‐79 (63)	42‐85 (63)
Total PSA ng/mL, median (IQR)	5.3 (3.93‐6.89)	4.36 (3.40‐6.01)	6.040 (4.87‐7.47)
%fPSA median (IQR)	0.169 (0.133‐0.231)	0.165 (0.133‐0.220)	0.170 (0.131‐0.240)
Prostate volume range, mL (median)	35‐250 (50)	35‐130 (49)	35‐250 (50)
No PCa n (%)	546 (57)	310 (64)	236 (50)
GG < 2, n (%)	239 (25)	107 (22)	132 (28)
GG ≥ 2, n (%)	170 (18)	64 (13)	106 (22)

Abbreviations: GG, grade group; IQR, interquartile range; PCa, prostate cancer; PSA, prostate‐specific antigen; %fPSA, percent free PSA.

### 3.2 Model development

The development of the Proclarix 5‐parameter biomarker model (herein referred to as biomarker model) incorporating THBS1, CTSD, tPSA, %fPSA and age was performed on all 955 samples to calculate specificity, NPV and PPV at a fixed sensitivity of 90% to identify significant cancer. For A 90% sensitivity, the cut‐off for the multivariate model was calculated at 10% with uncertainty of the cut‐off expressed as the 90% nonparametric confidence interval of 7 to 12%. Specificity of the model (at 90%) was 43% (95% CI 39%‐46%) with an NPV of 95% (95% CI 92%‐97%) and a PPV of 25% (95% CI 22%‐29%). This is in comparison to %fPSA alone which at a 90% sensitivity results in a specificity of only 17% (95% CI 14%‐20%). In addition, the NPV of 89% (95% CI 83%‐93%) and a PPV of 19% (95% CI 16%‐22%) is lower than the 5‐parameter model. Comparison of specificities of the 5‐parameter model and %fPSA alone at 90% sensitivity yielded a statistically significant result (McNemar Test *P* value < .001).

### 3.3 Model assessment: Split sample approach

**Figure 1:**
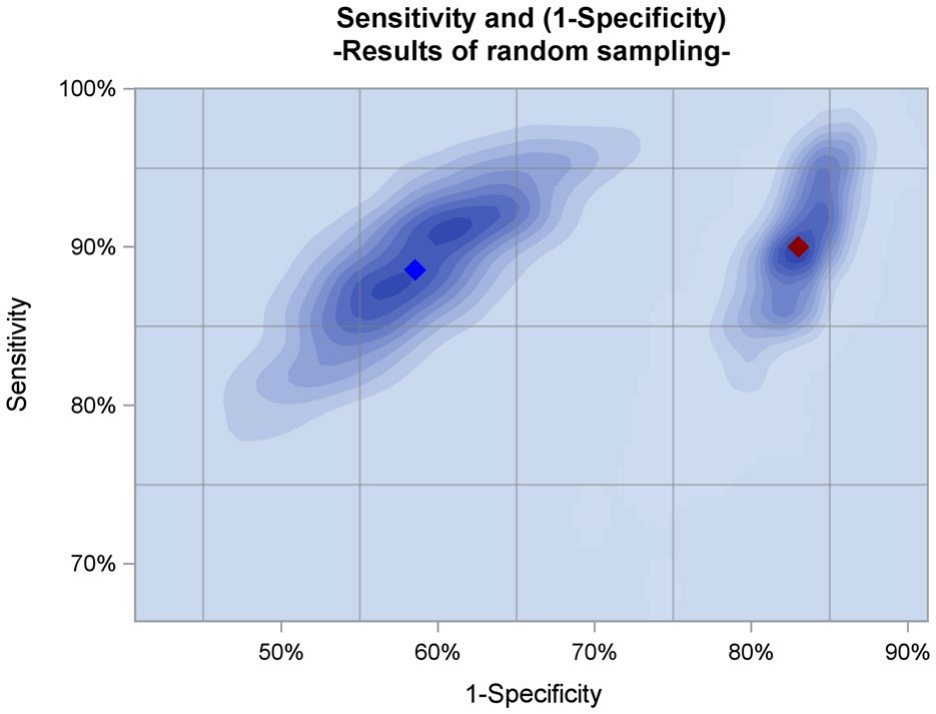
Summary of Proclarix and %fPSA alone validation using 1000 independent sets of random sampling. Proclarix (blue diamond) consistently demonstrates higher specificity across all sets at 90% sensitivity for significant PCa compared to %fPSA alone (red diamond)

**Figure 2:**
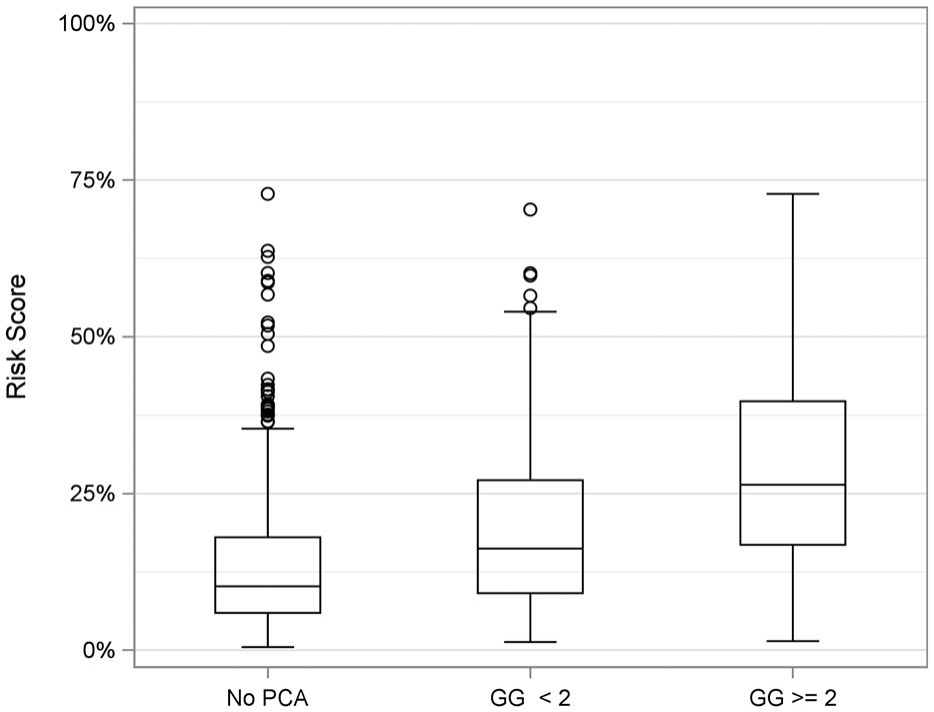
Risk score correlation with aggressiveness of PCa detected on biopsy. Boxplot shows an increasing risk score for more significant cancer. Kruskal–Wallis (*P* < .001*)*

Following development of the biomarker model, a split sample training‐validation approach was used to yield reliable performance predictions. The median specificity based upon 1000 independent sets of training and validation resulted at 89% sensitivity (derived from cut‐off at 90% from training set) in 42% specificity, NPV of 95% and PPV of 25%, respectively, for Proclarix. This is in comparison to %fPSA alone at 90% sensitivity which displayed a specificity of 17%, 89% NPV and 19% PPV respectively (Table 2 and Figure 1). As the test displayed a median specificity of 42% in 1000 independent validations, the biomarker model was shown to be validated with respect to its suitability in predicting clinically significant PCa. Values for training and validation for both the biomarker model and %fPSA were similar, suggesting limited overfitting of the model (Table 2). The Proclarix biomarker model results in a risk score that showed a significant increase across groups (no PCA, GG < 2 and GG ≥ 2) (Kruskal‐Wallis *P* < .001) and could thus differentiate aggressiveness of clinically significant PCa detected on biopsy (Figure 2).

**Table 2:** Comparison of performance characteristics of Proclarix and %fPSA at fixed sensitivity of 90% for clinically significant PCa

Performance characteristic	Proclarix		%fPSA	
	Training	Validation	Training	Validation
Sensitivity % (median)	90	89	90	90
Specificity % (median)	42	41	17	17
NPV % (median)	95	95	89	90
PPV % (median)	25	25	19	19

Note

Median values as obtained from 1000 independent sets of training and validation.

Abbreviations: NPV, negative predictive value; PPV, positive predictive value.

### 3.4 Model impact on biopsy decision

As the cut‐offs were calculated for a sensitivity of 90%, the accepted (by the design) rate of missed cancers within clinically significant PCa was 10%. This corresponded to 17 (out of 170) clinically significant PCa cases (Table 3), with 12 Gleason 3 + 4 (GG 2), two 4 + 3 (GG 3), two 3 + 5 and one 4 + 4 (GG 4), while no GG 5 cancers were missed. In comparison, using %fPSA as the decision support test, 13 Gleason 3 + 4 (GG 2), two 4 + 4 and two 3 + 5 (GG 4) were missed. The number of avoided biopsies would be more than double in the biomarker model (37%) compared to %fPSA (16%), while keeping the number of missed cancers constant.

**Table 3:** Number of missed cancers by grade group and Gleason score for biomarker model and %fPSA alone (90% sensitivity)

Grade group	2	3	4		5				Total
Gleason score	3 + 4	4 + 3	3 + 5	4 + 4	4 + 5	5 + 3	5 + 4	5 + 5	
Proclarix	12	2	2	1	0	0	0	0	17
%fPSA	13	0	2	2	0	0	0	0	17

## 4 DISCUSSION

Our study comprising of 955 men from two different cohorts shows that Proclarix can discriminate clinically significant PCa from other prostate conditions with a high specificity of 43% at 90% sensitivity in a particularly challenging patient segment with a PSA of 2‐10 ng/mL, prostate volume ≥35 mL and a normal DRE. The test displays a high NPV and PPV while limiting the number of missed cancers and clearly outperforms %fPSA alone (specificity of 17% at 90% sensitivity). The test validated in this study uses two novel biomarkers (THBS1 and CTSD) with patient age, tPSA and %fPSA. Importantly, it contains only objectively determinable input parameters and is thus not susceptible to arbitrarily determined parameters such as DRE or prostate volume. The glycoprotein markers have been identified previously using a genetic‐guided proteomics approach and have been shown to be effective in predicting prostate biopsy outcome. Further, in contrast to several other test approaches, these two novel biomarkers are independent of the different PSA isoforms and have a documented role in cancer development. Furthermore they are measured on standard laboratory equipment^20^ in serum from blood samples taken in the routine patient assessment procedure.^15^ Thus, the Proclarix test can be applied in any diagnostic laboratory.

The decision to use PSA for diagnosis of clinically significant PCa is notoriously challenging. Its use can result in high numbers of biopsies being performed but with low numbers of positive outcomes, whereby it is reported that up to 75% of the biopsies performed in the 4‐10 ng/mL range can be negative and are suggested to be un‐necessary.^21^ In addition, reported tPSA sensitivities and specificities can be dependent on the cohort used^22^ as well as DRE status.^23^ The multitude of available diagnostic tests can result in a *diagnostic grey zone* as an additional layer of complexity to the *PSA grey zone*, where the correct selection of diagnostic tools is a challenge, especially in complex cohorts. In addition, the diagnostic landscape is also changing with the implementation of multi‐parametric magnetic resonance imaging (mpMRI). However, indeterminate mpMRI cases are a common finding and those men who undergo biopsy have PCa in 12%‐33% of cases, of which 4%‐12% can be clinically significant.^24^ Further, in the case of a negative mpMRI, detection of clinically significant PCa found at systematic biopsy has been reported to be from 0% to 20%.^25^

Proclarix has been validated on a large cohort representative of the indications for use including cohorts from a screening centre as well as a referral centre. While the Martini‐Klinik in Hamburg is a typical referral centre, the Medical University Innsbruck is actively inviting men for PSA screening.^26^ The test combines clinically relevant serum biomarkers and demographic characteristics with a proprietary standalone software that packages the multivariate algorithm into a user interface to produce a risk score. Conclusively, we have shown that Proclarix could be used as an aid for informed decision making for prostate biopsy to identify clinically significant PCa in a challenging and growing patient population.

Within this study, the serum samples were collected prospectively, but were retrospectively analysed. All samples from the biobanks matching all required inclusion and exclusion criteria were used for the cohorts and all cases were consecutive to remove any bias. In this study, the prostate volume for the patients was obtained by TRUS and not only estimated by DRE, which would be the general practice. However, volume is only used as an inclusion criterion and not as an input for the model. In addition, neither family history of cancer nor race, factors known to have an influence on PCa,^27^ were included in the prediction model.

Cases explored with mpMRI have not been included in this validation study, and the use of Proclarix will be investigated in this situation; either in combination with mpMRI use, to select or screen patients prior to mpMRI, or in the cases where mpMRI is indeterminate or negative to confirm this result.

## 5 CONCLUSIONS

This study demonstrated that Proclarix, incorporating THBS1, CTSD, tPSA, %fPSA and age, reached a high level of clinical performance related to an increased specificity especially when compared to %fPSA alone. Proclarix represents an aid in deciding which subjects suspected of clinically significant PCa should undergo a prostate biopsy. At a sensitivity of 90% for clinically significant PCa, Proclarix has a specificity of 43% compared to 17% for %fPSA. The test has been CE marked and validated for subjects with a suspicion of PCa and a tPSA of 2‐10 ng/mL, prostate volume ≥35 mL, no prior history of PCa and a normal DRE. With a sensitivity of 90% and a specificity of 43%, it has the potential to lower the rate of negative prostate biopsies while accurately predicting clinically significant PCa in a very challenging and growing patient population. Ongoing multicentre clinical studies will expand on these results in additional cohorts and assess how the test could support mpMRI (PROPOSe trial: Identifier: NCT03565289, INNOVATE trial, Identifier: NCT02689271).

## CONFLICT OF INTERESTS

Some of the authors have received/held stock options (SG) and salaries (BG) and founder shares (RS) from ProteoMediX. SK and TS are advisors to ProteoMediX. TS and RS are inventors of the patent application WO2018011212, and SG and RS have the patent application 32 WO2009138392.

## FUNDING INFORMATION

Funding for this work was provided by ProteoMediX AG.
